# Randomized controlled study of the impact of a participatory patient care plan among primary care patients with common chronic diseases: a one-year follow-up study

**DOI:** 10.1186/s12913-021-06716-6

**Published:** 2021-07-20

**Authors:** Nina Tusa, Hannu Kautiainen, Pia Elfving, Sanna Sinikallio, Pekka Mäntyselkä

**Affiliations:** 1 Institute of Public Health and Clinical Nutrition, University of Eastern Finland, P.O. Box 1627, FI-70211 Kuopio, Finland; 2 Primary Health Care Unit, Kuopio University Hospital, Kuopio, Finland; 3 Siilinjärvi Health Center, Siilinjärvi, Finland; 4 Folkhälsan Research Center, Helsinki, Finland; 5 Department of Medicine, Kuopio University Hospital, Kuopio, Finland; 6 School of Educational Sciences and Psychology, University of Eastern Finland, Joensuu, Finland

**Keywords:** Primary health care, Patient care planning, Care plan, Treatment plan, Impact, Chronic diseases, Ageing population

## Abstract

**Backround:**

Chronic diseases and multimorbidity are common in the ageing population and affect the health related quality of life. Health care resources are limited and the continuity of care has to be assured. Therefore it is essential to find demonstrable tools for best treatment practices for patients with chronic diseases.

Our aim was to study the influence of a participatory patient care plan on the health-related quality of life and disease specific outcomes related to diabetes, ischemic heart disease and hypertension.

**Methods:**

The data of the present study were based on the Participatory Patient Care Planning in Primary Care. A total of 605 patients were recruited in the Siilinjärvi Health Center in the years 2017–2018 from those patients who were followed up due to the treatment of hypertension, ischemic heart disease or diabetes. Patients were randomized into usual care and intervention groups. The intervention consisted of a participatory patient care plan, which was formulated in collaboration with the patient and the nurse and the physician during the first health care visit.

Health-related quality of life with the 15D instrument and the disease-specific outcomes of body mass index (BMI), low density lipoprotein cholesterol (LDL-C), hemoglobin A1c (HbA1C) and blood pressure were assessed at the baseline and after a one-year follow-up.

**Results:**

A total of 587 patients with a mean age of 69 years were followed for 12 months. In the intervention group there were 289 patients (54% women) and in the usual care group there were 298 patients (50% women). During the follow-up there were no significant changes between the groups in health-related quality and disease-specific outcomes.

**Conclusions:**

During the 12-month follow-up, no significant differences between the intervention and the usual care groups were detected, as the intervention and the usual care groups were already in good therapeutic equilibrium at the baseline.

**Trial registration:**

ClinicalTrials.gov Identifier: NCT02992431. Registered 14/12/2016

## Backround

Patient engagement and activation in self-care, patient-centered care, self-management support and shared decision-making are important primary health care and health policy issues [1–4]. Populations around the world are rapidly ageing and the number of chronic diseases and long-term health problems is growing [5–7]. Health care resources are limited and the continuity of care has to be assured. One of the tools for answering this demand and gather information together is a care or a treatment plan. In Finland, the National Institute for Health and Welfare has issued a recommendation and guidance on the use of the structured care plan mentioned in the Health Care Act [8].

An individual or personalised care plan or a treatment plan is a detailed approach to care goals tailored to an individual patient’s needs. In this article, we use the term care plan. It is a mutual agreement between the patient and the health staff regarding the goals the patient is ready and willing to achieve to improve his health and well-being. It also describes what kind of support the patient needs and what the treatment targets are from a medical perspective [9].

Although various care plans have been used in Finland and elsewhere for decades, there is still little research on their effectiveness [8, 10–15]. Earlier Finnish care plan projects have reported a decrease in the number of health care visits in the long run and improvements in blood lipid levels, blood pressure levels and body mass index (BMI) [12, 16, 17]. In Australia (2009), the use of a care plan improved the treatment of depressed patients, reduced their 10-year risk of cardiovascular disease, and increased their physical activity [10, 18]. The care plan also appears to have the potential to shift the focus from the treatment of a single disease to acknowledging the treatment of the ensemble of all chronic diseases [10, 18]. According to the Cochrane Review, it has a small positive impact on diabetes management, blood pressure, asthma balance, depression, and self-care, but not on perceived health [19]. The impact of a care plan was found to be greater with more care plan rounds, more frequent patient contacts with health care, and when the physician treating the patient the most was involved in the process.

The pivotal shift in the care plan process is from reactive care to a proactive approach and patient-centred goal setting and action planning. Patients themselves, through their daily actions and choices, determine - to a large extent - their need for care and health care outcomes. Although there already is some research on self-management support strategies in primary care practices [4], it is not known how to support the patient best to achieve their own goals in real life. Moreover, more research is needed on how to find and motivate particularly those patients who are reluctant to invest in their well-being.

The health related quality of life (HRQOL) is an important outcome of the health care playing a notable role in patients with chronic diseases [20]. The HRQOL is dependent on the number of the chronic diseases and is higher with the lighter burden of diseases [21]. For example, hypertension and diabetes are experienced with a minor decrease in HRQOL than depression, cancer, asthma and chronic obstructive pulmonary disease [22, 23]. One purpose of the care plan is to support continuity which is particularly important in patients with chronic illnesses and improving the quality of care [24–29]. However, in the health care there is a continuous need in balancing with the resources and need. Therefore, we need more research on applying and implementing integrative methods of supporting the continuity and coordination of care.

The ageing population and increased prevalence of chronic diseases with the increased need for supporting continuity and involving patients in their health care are important reasons for the health care plan. The process of the health care plan ties the resources of different health care professionals. However, the benefits related to treatment and HRQOL among primary health care patients with common chronic diseases are vague. Therefore, in the present real-life randomized study, the aim was to investigate whether a participatory patient care planning intervention has any impact on the health related quality of life (HRQOL) and clinical outcomes in primary care patients with hypertension, ischemic heart disease or diabetes.

## Methods

### Context

The study was a real-life randomized control study integrated in the everyday work in a health center. The data of the present study were based on the Participatory Patient Care Planning in Primary Care (4PHC) (ClinicalTrials.gov Identifier: NCT02992431). The study was conducted in semi urban municipality Siilinjärvi, Finland, with a population of 21,657 residents at the end of 2017, located in the vicinity of a large town. The mean-age of the population in Siilinjärvi was 41 years in 2017 [30].

### Patients

The study population was based on adult residents (age ≥ 18 years) who were living in the municipality of Siilinjärvi, and had diabetes, ischemic heart disease or hypertension. They were registered in the electronic patient records in the Siilinjärvi Health Center. The Siilinjärvi Health Center is the main primary health service responsible for the health of the population in primary care in the municipality. The participating patients were recruited from those patients who had a follow-up visit due to their disease between February 2017 and March 2018. The disease groups were organized according to the degree of severity of the disease so that the hypertensive patients had only hypertension, ischemic heart disease patients could have also had hypertension and the diabetes patients could have had all three diseases. Of the diabetes patients, 58 had ischemic heart disease (24%) and 193 had hypertension (80%). Of the patients with ischemic heart disease, 59 (57%) had hypertension. In the diabetes group there were both type 1 and 2 diabetes patients. In addition to these diseases, patients could also have other diseases. The baseline results have been presented in more detail in our previous article [31].

**Fig. 1:**
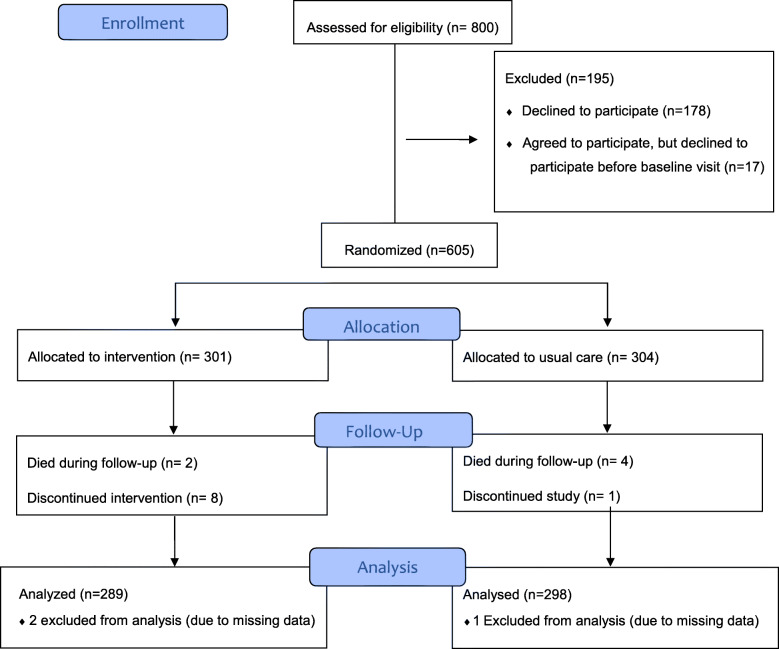
Flow Diagram of the study

A total of 800 patients were informed about the study. In total, 622 patients agreed to participate, but 17 cancelled their participation afterwards. Of the 605 participants, 587 patients completed all the data needed for this analysis. The flow of the study is presented in Fig. 1.

### Randomization

The participants were randomly assigned to an intervention group or a control group receiving usual care (UC). The randomization was stratified according to disease and was carried out by a statistician outside the research team. The invitation letters in sealed envelopes were arranged according to the randomization. Before the scheduled appointment, the study nurse sent the concealed invitation letter to the patients, who had given a written consent to the study. It was not possible to blind participants, health professionals or researchers due to the nature of the intervention.

### Intervention

**Fig. 2:**
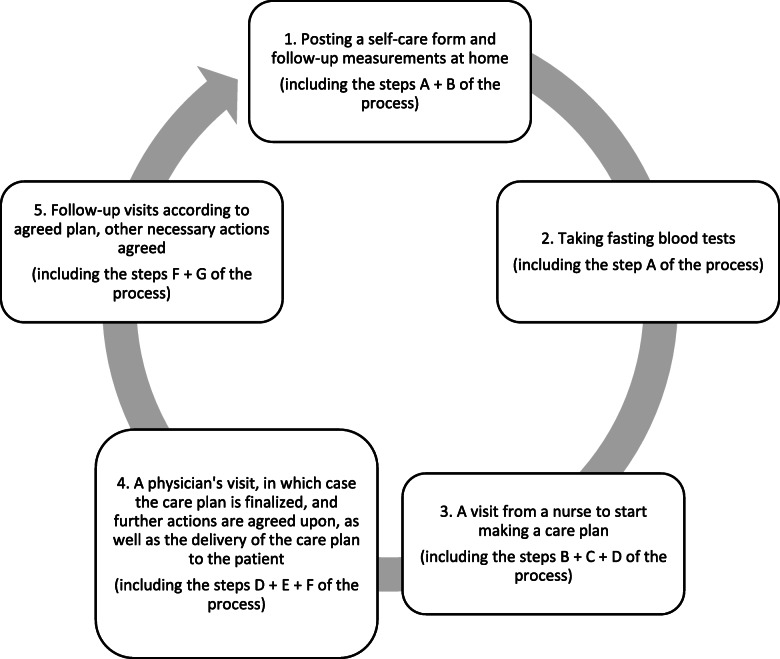
The participatory care plan process. The process steps are preparation (**A**), goal setting (**B**), action planning (**C**), documenting (**D**), coordinating (**E**), supporting (**F**) and reviewing (**G**)

Intervention consisted of the participatory patient care plan (PPCP). It included the patient activation questionnaire form and a request to attach records of self-monitored measurements, such as blood sugar and blood pressure values. After advance preparation, the patient was invited to visit the nurse and a general practitioner and a participatory care plan was mutually accepted by the patient and the health staff. All medical staff received short training on the preparation of a PPCP and a written instruction on the research protocol was distributed to each office, which also included a care plan formulation guideline. At the nurse visit, each patient had an appointment for 30–60 min in which the patient and the nurse started the discussion about his or her lifestyle, individual goals for the treatment and conducted the measurements (blood pressure in sitting position, waist measurement, weight and length). A fasting blood sample was drawn in a laboratory after 12 h of fasting before the physician’s visit. Then the patient visited the general practitioner for 30–40 min for the follow-up and discussion about the treatment goals and follow-up plan resulting in the written PPCP. Briefly, the personalized care plan process includes the following steps as presented at Fig. 2: preparation (A), goal setting (B), action planning (C), documenting (D), coordinating (E), supporting (F) and reviewing (G) [9].

See detailed description of the PPCP in Table 1.

**Table 1:** Participatory patient care plan framework themes

Theme	Description
Need for care	Narrative description of the need for care (patient perspective)
Goal of treatment	Narrative description of the goals of treatment (patient perspective)
Treatment implementation and means	Narrative description of the implementation and means of treatment
Support, monitoring and evaluation	Narrative Description of support for the realization of the patient care plan, monitoring and assessment of the effects of treatment
Medication	The current medication
Patient care plan delivery	Deliver a care plan to the patient**.** The care plan is mailed to the patient by post or he/she can view it My Kanta digital services for browsing his/her medical records and prescriptions.

### Usual care

Participants in the UC group had the same measurements as the intervention group in a nurse’s office and at the same visit they met the general practitioner (GP) or the GP phoned a follow-up call.

### Outcomes

Primary outcomes were health-related quality of life (HRQOL) measured with the 15D [32], and disease-specific outcomes: blood pressure, glycemic control measured with hemoglobin A1c (HbA1C), dyslipidemia treatment status measured with low-density lipoprotein cholesterol (LDL-C) and BMI.

The 15 D is a generic, comprehensive, standardized, self-administered measure of health-related quality of life. The 15D questionnaire includes the following 15 dimensions: breathing, mental function, speech (communication), vision, mobility, usual activities, vitality, hearing, eating, elimination, sleeping, distress, discomfort and symptoms, sexual activity and depression. Each dimension is divided into 5 levels. The maximum score is 1 (no problems on any dimension) and the minimum score is 0 (being dead). The smallest change in quality of life detected by the 15D is 0.015 [33].

A fasting blood sample for HbA1C and LDL-cholesterol was drawn in a laboratory after 12 h of fasting. The standard procedure of the Kuopio University Hospital laboratory was used in the analysis.

A trained nurse measured blood pressure in a sitting position after 10 min of sitting. Diastolic blood pressure (DBP) and systolic blood pressure (SBP) were recorded and mean arterial pressure (MAP) was calculated [DP+ 1/3(SBP-DBP)]. The nurse also measured weight in light clothing and height, and the body mass index was calculated as weight (kg)/height (m)^2^.

### Other measurements

Educational background and relationship status were asked in the questionnaire. Accordingly, the presence of other chronic diseases was asked. Depressive symptoms were measure with the 21-item Beck’s Depression Inventory [34]. The number of drinks per week and current smoking (yes or no and number of cicarettes per day) habits were asked in the question form.

Physical activity was measured by the Kasari fit index [35]. The FIT index was used to assess the level of physical activity and was calculated according to the participant’s responses on an activity questionnaire scale: [FIT = Frequency (F) x Intensity (I) x Time (T)]. The participant’s frequency was determined by how many days per week he/she exercised on a five-point scale with 1 representing less than once per month and 5 representing 6 or 7 times per week. Intensity was set on a five-point scale with 1 representing light aerobic activity such as normal walking and 5 representing high intensity such as running. Time was determined on a four point scale with 1 representing less than 10 min and 4 representing greater than 30 min. FIT scores can range from 1 (low activity) to 100 (high activity) and are divided into four classes 0–12 points, 13–36 points, 37–63 poits and over 64 points [36].

### Statistical methods

Sample size calculations were based on the 15D, primary outcome measure. A 0.015 change in this score has been considered a clinically meaningful difference between intervention and control arms [33]. To ensure 85% power to detect this difference in the 15D with type 1 error 5% size (=0.05, power = 85%), the sample size would be 300 per group. We expected the drop-out rate over time to be about 10%.

The intention-to-treat analysis was applied in all analyses. Data were presented as means with standard deviation (SD) and as counts with percentages. Statistical comparisons between groups were done using a t-test and Chi-square test. The adjusted mean changes of outcome measures between the baseline and 12-month measures were assessed using analysis of covariance (ANCOVA), with baseline measure as covariates. Effect sizes (Cohen’s d) were calculated to determine the magnitude of the difference between the means of the groups. Effect sizes of 0.20, 0.50 and 0.80 were considered small, medium, and large, respectively [37, 38]. Confidence intervals (CIs) for the effect sizes were obtained by bias-corrected bootstrapping (5000 replications). The normality of the variables was evaluated graphically and by the Shapiro–WilkW test. The Stata 16.1 (StataCorp LP; College Station, Texas, TX, USA) statistical package was used for the analysis.

All methods were carried out in accordance with relevant guidelines and regulations.

The study protocol was approved by the Research Ethics Committee of the Northern Savo Hospital District (410/2016). ClinicalTrials.gov Identifier: NCT02992431. Registered 14/12/2016.

## Results

There were 289 patients (54% women) in the intervention group and 298 patients (50% woman) in the usual care group. The mean age in both groups was 69 years. The basic charasteristics of the two study groups are presented in Table 2.

**Table 2:** Characteristics of the patients

	Intervention	Usual care	*P*-value
	*N* = 289	*N* = 298	
Women, n (%)	157 (54)	150 (50)	0.33
Age, mean (SD)	69 (9)	69 (9)	0.35
Living with a spouse, n (%)	215 (74)	215 (72)	0.54
Number of education years, mean (SD)	10.1 (2.9)	10.5 (3.3)	0.63
Retired, n (%)	239 (83)	256 (86)	0.29
Smoking, n (%)	32 (11)	29 (10)	0.59
Alcohol consumption, n (%)			0.65
Not at all	67 (23)	81 (27)	
one times per month or less	101 (35)	87 (29)	
2–4 times per month	76 (27)	91 (31)	
2–3 times per month	31 (11)	30 (10)	
4 or more times per month	11 (4)	7 (2)	
Moves without mobility aids, n (%)	264 (91)	270 (91)	0.75
Physical activity^a^, mean (SD)	41 (20)	40 (20)	0.44
Fasting plasma glucose, mmol/l, mean (SD)	6.55 (1.19)	6.61 (1.33)	0.58
Beck depression index score, mean (SD)	5.9 (4.9)	5.7 (4.9)	0.56
Diseases, n (%)			
Diabetes mellitus	124 (42.9)	117 (39.3)	0.37
Hypertension	230 (79.6)	246 (82.6)	0.36
Ischemic heart disease	77 (26.6)	86 (28.9)	0.55
Atrial fibrillation	38 (13.1)	33 (11.1)	0.44
Cardiac failure, insufficiency	18 (6.2)	21 (7.0)	0.69
Musculoskeletal disorders	155 (53.6)	157 (52.7)	0.82
Dementia	5 (1.7)	2 (0.7)	0.28
Other neurological diseases	14 (4.8)	14 (4.7)	0.99
Depression or other psychiatric disorders	21 (7.3)	14 (4.7)	0.22
Asthma or chronic obstructive pulmonary disease	39 (13.5)	40 (13.4)	0.98
Cancer	3 (1.0)	1 (0.3)	0.37
Number of diseases, mean (SD)	2.5 (1.3)	2.5 (1.2)	0.64

*%* percentage, *SD* standard deviation, *n* number

^a^Kasari FIT index

After a 12-month follow-up, there was a significant decrease in systolic blood pressure and mean arterial pressure in the usual care and intervention groups when all participants were assessed together (Table 3). There were no significant differences in the changes of the 15D, BMI, LDL-C, HbA1C or blood pressure between the intervention group and usual care group.

**Table 3:** Baseline values and changes after 12 months of outcome measures of patients in intervention and usual care groups

	Baseline		Change		*P*-value**	
	Intervention Mean (SD)	Usual care Mean (SD)	Intervention Mean (95% CI)	Usual care Mean (95% CI)	Crude	Adjusted^a^
**ALL**						
HRQOL, 15D score	0.867 (0.095)	0.876 (0.093)	0.004 (−0.003 to 0.01)	− 0.002 (− 0.009 to 0.005)	0.26	0.42
Body mass index, kg/m2	29.2 (5.2)	29.5 (5.8)	− 0.17 (− 0.35 to 0.01)	− 0.04 (− 0.19 to 0.11)	0.28	0.25
LDL-C, mmol/l	2.67 (0.97)	2.59 (0.92)	−0.01 (− 0.09 to 0.09)	0.06 (− 0.02 to 0.14)	0.32	0.45
HbA1C, mmol/mol	40.9 (8.2)	41.8 (8.8)	0.4 (−0.1 to 1.0)	0.1 (−0.5 to 0.8)	0.46	0.83
Blood pressure (mmHg)						
Systolic	148 (17)	148 (18)	−3.3 (−5.6 to −1.0)	−2.5 (−4.5 to − 0.5)	0.62	0.64
Diastolic	82 (11)	82 (10)	−1.1 (−2.4 to 0.1)	− 1.1 (− 2.2 to 0.0)	0.94	0.90
Mean arterial pressure	103 (11)	103 (11)	−1.9 (−3.3 to −0.4)	−1.6 (− 2.8 to − 0.3)	0.75	0.74

*SD* standard deviation, *HRQOL* health-related quality of life, *LDL-C* low-density lipoprotein cholesterol, *HbA1C* Haemoglobin A1c

** *P*-value for the differences in the changes between the intervention group and usual care group

^a^ANCOVA: adjusted for baseline value

Table 4 shows the disease-specific changes. In patient with hypertension, HbA1C increased and blood pressure decreased in both groups. In patients with ischemic heart disease, HbA1C increased in both groups. In patients with diabetes, BMI decreased in the intervention group. There were no significant differences in the changes in the 15D, BMI, LDL-C, HbA1C or blood pressure between intervention and usual care in any of the the disease groups.

**Table 4:** Baseline values and change after 12 months of outcome measures in intervention and usual care groups for the patients having hypertension, ischemic heart disease and diabetes

	Baseline		Change		*P*-value**	
	Intervention Mean (SD)	Usual care Mean (SD)	Intervention Mean (95% CI)	Usual care Mean (95% CI)	Crude	Adjusted^a^
**Hypertension**						
HRQOL, 15D score	0.880 (0.083)	0.891 (0.082)	0.007 (−0.004 to 0.017)	− 0.001 (− 0.011 to 0.10)	0.34	0.52
Body mass index, kg/m2	27.9 (4.6)	27.9 (5.0)	0.06 (−0.24 to 0.35)	−0.04 (− 0.24 to 0.16)	0.59	0.58
LDL-C, mmol/l	3.04 (0.95)	2.95 (0.93)	−0.07 (− 0.23 to 0.09)	0.05 (− 0.10 to 0.20)	0.27	0.37
HbA1C, mmol/mol	36.8 (4.3)	37.9 (3.6)	0.6 (0.1 to 1.0)	0.3 (0.1 to1.0)	0.41	0.98
Blood pressure (mmHg)						
Systolic	144 (16)	144 (17)	−4.2 (−8.0 to −0.4)	− 4.0 (−7.1 to −1.0)	0.95	0.65
Diastolic	83 (10)	85 (11)	−2.0 (−4.0 to −0.0)	− 2.2 (−3.9 to − 0.5)	0.91	0.64
Mean arterial pressure	105 (11)	106 (11)	−2.7 (−5.1 to −0.4)	−2.8 (− 4.7 to − 0.9)	0.97	0.62
**Ischemic heart disease**						
HRQOL, 15D score	0.860 (0.085)	0.860 (0.106)	0.001 (−0.014 to 0.015)	−0.012 (− 0.029 to 0.005)	0.28	0.28
Body mass index, kg/m2	28.1 (4.4)	28.3 (5.5)	−0.40 (− 0.87 to 0.08)	−0.20 (− 0.49 to 0.09)	0.47	0.40
LDL-C, mmol/l	2.21 (0.74)	2.23 (0.80)	0.09 (−0.09 to 0.27)	0.05 (−0.12 to 0.22)	0.77	0.78
HbA1C, mmol/mol	38.5 (3.9)	38.3 (4.7)	0.7 (0.1 to 1.4)	0.3 (−0.4 to 1.0)	0.40	0.31
Blood pressure (mmHg)						
Systolic	144 (19)	143 (16)	−3.1 (−7.5 to 1.3)	−0.1 (−4.4 to 4.3)	0.33	0.87
Diastolic	82 (11)	79 (9)	−1.5 (−4.2 to 1.2)	−0.7 (−3.3 1.9)	0.65	0.83
Mean arterial pressure	103 (11)	100 (10)	−2.1 (−4.8 to 0.7)	−0.5 (−3.2 to 2.2)	0.42	0.74
**Diabetes mellitus**						
HRQOL, 15D score	0.856 (0.108)	0.868 (0.096)	0.002 (−0.010 to 0.013)	0.001 (−0.010 to 0.012)	0.91	0.84
Body mass index, kg/m2	31.0 (5.5)	31.6 (6.2)	−0.30 (− 0.54 to − 0.07)	0.03 (− 0.26 to 0.32)	0.081	0.078
LDL-C, mmol/l	2.49 (0.94)	2.38 (0.83)	0.03 (−0.10 to 0.15)	0.07 (−0.04 to 0.19)	0.61	0.74
HbA1C, mmol/mol	45.6 (9.7)	46.7 (10.9)	0.2 (−0.8 to 1.3)	−0.1 (−1.4 to 1.3)	0.75	0.91
Blood pressure (mmHg)						
Systolic	145 (19)	144 (17)	−2.4 (−6.1 to 1.3)	1.9 (−5.4 to 1.5)	0.86	0.54
Diastolic	81 (12)	80 (10)	−0.1 (−2.1 to 1.9)	−0.1 (−1.9 to 1.6)	0.97	0.81
Mean arterial pressure	102 (12)	101 (10)	−0.9 (−3.2 to 1.4)	− 0.7 (−2.9 to 1.4)	0.94	0.89

*SD* standard deviation, *HRQOL* health-related quality of life, *LDL-C* low-density lipoprotein cholesterol, *HbA1C* Haemoglobin A1c

** *P*-value for the differences in the changes between the intervention group and usual care group

^a^ANCOVA: adjusted for baseline value

**Fig. 3:**
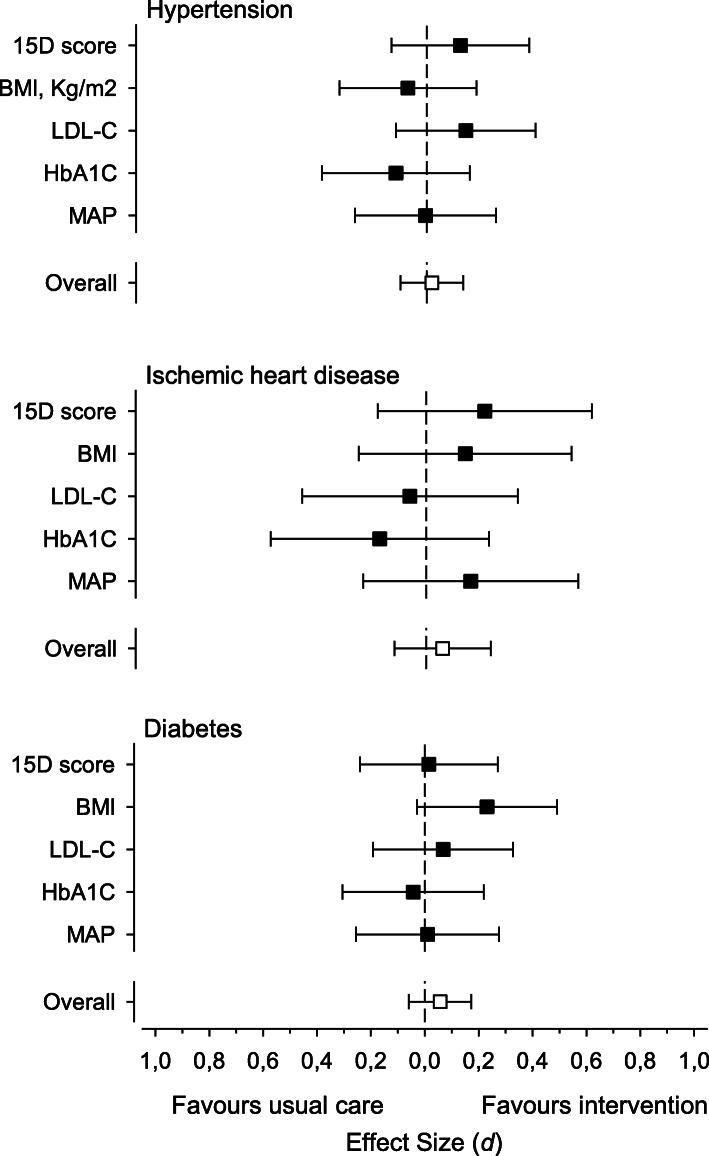
Effect sizes (Cohen’s d) were calculated to determine the magnitude of the difference between the means of the three disease groups in health-related quality of life (15D), body mass index (BMI), low-density lipoprotein cholesterol (LDL-C), Hemoglobin A1c (HbA1C), mean arterial pressure (MAP) and overall. Effect sizes of 0.20, 0.50 and 0.80 were considered small, medium, and large, respectively. Adjusted for baseline value

In Fig. 3, the difference in change between the two groups is presented by Cohen’s effect size. There were no significant changes in the 15D, BMI, LDL-C, HbA1C or MAP in any disease group between the intervention and the usual care, athough change in BMI favoured slightly the intervention in patients with diabetes.

## Discussion

The intervention group did not seem to benefit from the participating patient care plan significantly after a twelve-month follow-up compared with the usual care group in any disease groups measured with health-related quality of life (HRQOL) or in disease-specific outcomes. There was a slight decrease in the body mass index within the diabetes group but not in the usual care at the twelve-month follow-up although the difference between intervention and usual care was not quite significant. In general, only minor (or no) changes were detected within groups.

We found few studies after the Cochrane review in addition to our study that have a follow-up while measuring the impact of care plans. One study concerned the diseases studied in our study. An observational, retrospective study of type 2 diabetes patients in primary care with mean follow-up time of 14 months showed statistical significant decrease in LDL-C, blood pressure and BMI [16]. A telephone interview study with female asthma patients after a two-year follow-up showed greater medication adherence and satisfaction with clinical care among patients having a negotiated care plan, but no significant associations between a negotiated care plan, asthma control and urgent care use [15]. In a primary care-based 1 year follow-up study in Australia, the use of a care plan improved the treatment of depressed patients, reduced their 10-year risk for cardiovascular disease, and increased their physical activity [10, 18]. This study was The TrueBlue model of collaborative care using practice nurses as case managers, the care plan being not the only intervention used to improve the care of depressive patients alongside diabetes or heart disease. An implementation study from the UK with primary care patients did not show a significant difference in mean vitality after a one-year follow-up [14]. Our findings are in line with these studies although in the Australian study, the influence was greater maybe partly due to the enhanced visits to the practice nurse and general practitioner every 3 months during the twelve-month follow-up. In our study, the patients continued their individually planned follow-up with their physician after 12–24 months or the nurse after 6–12 months depending on their individual need.

The trend of our findings is parallel with earlier research on diabetes management as it displayed a small positive impact on BMI although not significant on diabetes management. According to the Cochrane Review, the care plan has a small positive impact on diabetes management, blood pressure, asthma balance, depression, and self-care, but not on perceived health [19]. The impact of the care plan was found to be greater with more care plan rounds, more frequent patient contacts with health care, and if there was continuity with the same physician in the care plan process. In a Finnish cross-sectional study, a personalized care plan was positively associated with better clinical outcomes in type 2 diabetes patients such that patients who had a copy of their care plan were significantly more likely to achieve the systolic blood pressure target and low-density lipoprotein target and to use statins [12].

Our study did not find any significant changes of HRQOL. In the present study with 12 month follow-up, there may be several issues related to the small changes of HRQOL. Previously a Finnish population based study indicated that decrease of HRQOL is more significant in diabetes than in coronary heart disease or hypertension [20]. In cardiovascular diseases, older patients seem to experience less decrease in HRQOL compared with healthy ones [23]. It is plausible that in the present study, the patients with ischemic heart disease had quite stable treatment situation which is reflected by a low drop-out rate. In older diabetes patients, the higher HRQOL and better HbA1C balance have been associated [39]. In our study, diabetics had a good treatment balance on average. Hypertension, and especially the awareness of hypertension is known to slightly decrease the HRQOL [40, 41] although our study included only patients with known hypertension.

The earlier studies have shown a possible tendency to make care plans for patients with a worse therapeutic balance, which can affect the efficiency of the care plan [11, 15]. In our study the patients were randomized and there was no selection according to therapeutic balance. Their therapeutic balance was good, particularly in diabetic patients’ HbA1C, although BMI, LDL-C and blood pressure could be better in all the disease groups. However, the therapeutic balance was so good that improvement in any part of this kind of patient material would be quite challencing [42]. We can also consider these patients proactive because they were already registered and followed up in the health center and they were put in the automatic electronic health record calling system due to their chronic diseases. Thus, it is possible that part of the general goals of a care plan have already been achieved in the Finnish primary care, which can make it difficult to achieve significant further improvements with one intervention.

It seems that having a participatory care plan and being involved in the care plan process make patients more involved in their care and make their treatment more patient-centred [3, 11, 13, 14]. This can lead to improved self-efficacy and better control of the clinical outcomes in the long run. However, given the relatively short follow-up in our study, the results may improve with more care plan rounds in the future. Twelve months is a comparatively short time for older patients to change their lifestyles and maintain new patterns of self-care. Many of them have suffered from their long-term diseases for a long time and have already adjusted their lifestyles to the disease. In this study, the intervention protocol and the UC were clearly different, but it is also possible that some physicians have made some changes in their practice and interaction with their patients in the UC group as well.

The strength of this study was that it was a randomized controlled study with a follow-up. The patients in our study were on average almost 70 years old with an average of more than two chronic diseases. Thus, they seem to represent the patients of Finnish primary care well. The number of patients was considerably high, and the dropout rate was low. The study was implemented in the everyday real-life work in the health center and consisted of the general patient groups treated in primary care. The study used real follow-up data, not only self-reports and also considered clinical outcomes. There are some limitations of this study that need to be acknowledged also. We did not know the duration of the diseases and if the patients who agreed to participate were more interested in taking care of themselves than patients that refused to participate. Although the patients seemed to be quite representative of primary care patients, the study was conducted in one health center, which may not warrant unconditional direct generalization of the results to other parts of Finland or internationally.

To better understand the impact of the participatory care planning, we need more knowledge of the changes in clinical measures with a longer follow-up among multimorbid patients. We also need to explore the ideas and attitudes that both the patients and health staff have on care plans and the whole care plan process.

We conclude that among the primary care patients with hypertension, ischemic heart disease or diabetes, treatment with a participating patient care plan was not significantly superior compared with usual care after a twelve-month follow-up. The follow-up period might have been short for this kind of intervention and setting, suggesting the need for a longer follow-up.

## Data Availability

The data of the current study are not publicly available due to protection of individual privacy but are available from the corresponding author on reasonable request.

## Abbreviations

ANCOVA: Analysis of covariance
BMI: Body mass index
CI: Confidence index
DBP: Diastolic blood pressure
HbA1C: Haemoglobin A1c
HRQOL: Health-related quality of life
LDL-C: Low-density lipoprotein cholesterol
MAP: Mean arterial pressure
n: Number
PPCP: Participatory patient care plan
SBP: Systolic blood pressure
SD: Standard deviation
%: Percentage
UC: Usual care
